# Recent Developments in Lanthanide-Doped Alkaline Earth Aluminate Phosphors with Enhanced and Long-Persistent Luminescence

**DOI:** 10.3390/nano11030723

**Published:** 2021-03-13

**Authors:** Doory Kim

**Affiliations:** 1 Department of Chemistry, Hanyang University, Seoul 04763, Korea; doorykim@hanyang.ac.kr; 2 Research Institute for Convergence of Basic Sciences, Hanyang University, Seoul 04763, Korea; 3 Institute of Nano Science and Technology, Hanyang University, Seoul 04763, Korea; 4 Research Institute for Natural Sciences, Hanyang University, Seoul 04763, Korea

**Keywords:** lanthanide doping, strontium aluminates, calcium aluminates, barium aluminates, synthesis, phosphors, long-persistent luminescence, phosphorescence

## Abstract

Lanthanide-activated alkaline earth aluminate phosphors are excellent luminescent materials that are designed to overcome the limitations of conventional sulfide-based phosphors. The increasing research attention on these phosphors over the past decade has led to a drastic improvement in their phosphorescence efficiencies and resulted in a wide variety of phosphorescence colors, which can facilitate applications in various areas. This review article discusses the development of lanthanide-activated alkaline earth aluminate phosphors with a focus on the various synthesis methods, persistent luminescence mechanisms, activator and coactivator effects, and the effects of compositions. Particular attention has been devoted to alkaline earth aluminate phosphors that are extensively used, such as strontium-, calcium-, and barium-based aluminates. The role of lanthanide ions as activators and coactivators in phosphorescence emissions was also emphasized. Finally, we address recent techniques involving nanomaterial engineering that have also produced lanthanide-activated alkaline earth aluminate phosphors with long-persistent luminescence.

## 1. Introduction

Phosphorescence involves the emission of light for significant periods of time, even after removal of the exciting radiation. Unlike fluorophores, phosphors can store the absorbed light energy and release it as long-persistent luminescence in the form of a delayed weak radiation via forbidden energy state transitions. Such persistent, luminescent, and phosphorescent materials with an adequate lifetime have attracted considerable attention for a wide range of applications, such as devices based on organic light-emitting diodes (OLEDs) and in solar cells for energy conservation and emergency lighting.

Natural phosphorescence was first observed by Cellini in diamond in 1568, and several natural minerals have also been reported to generate similar emissions of light under illumination with sunlight, such as naturally doped willemite, scheelite, and calcite crystals [1]. The first artificial phosphor was synthesized via calcination of sulfur-rich barium sulfate in 1604 [2]. Sulfide-based phosphors, such as rare-earth alkali sulfides (CaS, SrS) and zinc sulfides doped with copper and cobalt (ZnS:Cu, ZnS:Co), were subsequently synthesized in the first half of the 20th century [3]. Among these, copper-doped zinc sulfide (ZnS:Cu) phosphors have been extensively employed as long-lasting phosphorescent materials in various areas, such as flat panel displays, cathode ray tubes, fluorescent lamps, and traffic signs [4]. Its afterglow intensity is known to be enhanced by doping with transition metals (such as cobalt) and radioactive elements (such as promethium); however, the mechanical and physical properties of the host are degraded when a large amount of a dopant is incorporated, which eventually leads to the rapid degradation of its chemical stability [5]. In addition, the low emission intensity and short intrinsic time of decay (1 h) of ZnS:Cu limit its applications [5].

Rare-earth-doped alkaline earth aluminates have been developed as potential phosphors to overcome such limitations. When alkaline earth aluminates (MAl_2_O_4_) are doped with rare-earth ions, the resulting structures can exhibit persistent luminescence for significantly long durations under sunlight illumination, thereby leading to them being considered as excellent luminescent materials [6,7,8,9,10]. Rare-earth elements are a group of 17 elements consisting of lanthanides, yttrium (Y), and scandium (Sc). Europium-doped strontium aluminates (SrAl_2_O_4_:Eu^2+^) were synthesized and studied as the first rare-earth-doped strontium aluminates in the late 1960s [11]. This development can be considered a breakthrough in the field of phosphorescence in terms of the applications of phosphor, primarily because of the high luminescence efficiency of such phosphors, which was ~10 times that of zinc sulfide; such phosphors also exhibited high chemical and physical stability [12]. Moreover, these phosphors are environmentally friendly, thanks to their low chemical toxicities and the lack of radioactive elements [13]. Particularly, the development of codoped SrAl_2_O_4_:Eu^2+^,Dy^3+^ (Dy: dysprosium) phosphors by Matsuzawa et al. has received considerable attention for the replacement of traditional ZnS-based phosphors, owing to their improved afterglow intensities, lifetimes, and chemical stabilities compared to those of the previously synthesized phosphors [5]. The intense emission of rare-earth-based phosphor has led to extensive applications in light-emitting diode (LED) devices, thin-film electroluminescent (TFEL) devices, optoelectronic or cathodoluminescent devices, safety marks, radiation dosimetry, X-ray imaging, bioimaging, and photodynamic therapy [14,15,16,17,18,19]. In particular, their applications as LED devices can help in replacing argon-mercury discharge fluorescent lamps, which are extensively used for general lighting purposes. They can also be applied as alternative mercury-free excitation sources for avoiding the use of hazardous ingredients and environmentally unsafe materials [20]. Moreover, they have been recently demonstrated as solar cell materials and high upconversion luminescent materials [21,22].

The phosphorescence from such lanthanide-activated alkaline earth aluminate phosphors can be tuned over the emission wavelength and lifetime, based on the 4f–5d transitions of lanthanides. The 4f–5d optical transition is electric-dipole allowed and, therefore, generally features a high radiative emission probability and short lifetime (of the order of tens of nanoseconds) [23,24,25]. This transition generates significantly broader absorption and emission spectra because of the high sensitivity of the 5d orbital to the surrounding environment. The luminescence properties arising from the 4f–5d transition of lanthanides can be tuned by varying the host material and lanthanide emitters. In particular, the luminescence properties arising from the 4f–5d transition of Eu^2+^ have been investigated in more than 300 compounds, and various emission colors from near-ultraviolet to deep red have been revealed [26].

Therefore, in this review, lanthanide-doped alkaline earth aluminate phosphors are discussed in the context of their potential as excellent luminescent materials. Lanthanide-doped strontium aluminate-, calcium aluminate-, and barium-aluminate-based phosphors are focused on, and their synthesis techniques, phosphorescence mechanisms, and effects of dopants and codopants are reviewed. Recent advances in the synthesis of lanthanide-doped alkaline earth aluminate phosphors are also highlighted. Finally, we discuss the prospects and challenges of the future development of lanthanide-doped alkaline earth aluminate phosphors.

## 2. Strontium Aluminate Phosphors

Strontium aluminates are the most extensively used alkaline earth aluminates and are convenient host crystals for rare-earth dopants [27]. They have attracted recent research interest that has resulted in their extensive applications in several fields owing to their excellent phosphor properties such as high brightness, long-persistent luminescence, and good chemical stability compared to those of other phosphor materials (Table 1). There are various types of strontium aluminate hosts, such as SrAl_2_O_4_, SrAl_2_B_2_O_7_, SrAl_4_O_7_, SrAl_12_O_19_, Sr_3_Al_2_O_6_, and Sr_4_Al_14_O_25_, all of which have different structures. For example, SrAl_2_O_4_ has a tridymite structure, whereas SrAl_12_O_19_ has a magnetoplumbite structure [28,29]. Among these, SrAl_2_O_4_ is a promising host material for ensuring persistent luminescence and acts as a convenient host crystal for rare-earth and transition metal dopants [30].

### 2.1. Lanthanide Ion-Doped Strontium Aluminate Phosphors

Eu^2+^ has been frequently used as an activator dopant in SrAl_2_O_4_ because it exhibits anomalously long phosphorescence. Because the Sr^2+^ and Eu^2+^ ions have similar ionic radii (1.21 and 1.20 Å, respectively), Eu^2+^ ions are very likely to be located in the Sr^2+^ positions; this was also confirmed by electron paramagnetic resonance (EPR) measurements [40,54]. Therefore, when Eu^3+^ ions are incorporated in the Sr^2+^ sites of SrAl_2_O_4_, they are observed to be easily reduced to Eu^2+^ [54,55]. In fact, two different sites are available for Sr ions to occupy, and they are slightly different with respect to the individual Sr–O distances (Sr^+^ and Sr^2+^ sites are coordinated at 6 and 7, respectively) [53]. The incorporation of Eu^2+^ ions in these different sites leads to a variation in the luminescent properties from both sites because of their different symmetries and orientations. The incorporated dopant ions are thought to introduce localized states of the bandgap in the host matrix, which facilitate the luminescent properties that arise from the various dopant-incorporated sites in the SrAl_2_O_4_ host material [56]. The doping sites in the host material are known to be determined by the host lattice components and the ionic radii of the dopants. For example, Eu^2+^, Eu^3+^, and Dy^3+^ are likely to be incorporated in the Sr^2+^ sites in the SrAl_2_O_4_ matrix because of their similar ionic radii^+^ (Eu^2+^: 1.2Å, Eu^3+^: 0.95 Å, Dy^3+^: 0.91 Å, Sr^2+^: 1.18 Å); the dissimilar ionic radii of Al^3+^ and O^2-^, compared to those of the dopants, ensure that the incorporation does not occur in their sites (Al^3+^: 0.53 Å, O^2-^: 1.4 Å). This was confirmed by EPR measurements [56].

**Figure 1:**
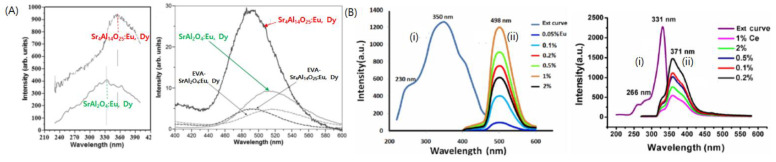
Photoluminescence spectra for lanthanide doped strontium aluminate phosphors (**A**) Photoluminescence excitation (left) and emission (right) spectra for SrA_2_O_4_:Eu^2+^,Dy^3+^ and Sr_4_Al_14_O_25_:Eu^2+^,Dy^3+^. Reprinted from [61] with permission from Elsevier. (**B**) Photoluminescence excitation (i) and emission (ii) spectra for SrA_2_O_4_:Eu^2+^ (left) and SrA_2_O_4_:Ce^3+^ (right). Adapted from [30] under Creative Commons Attribution (CC BY) license.

When the SrAl_2_O_4_ host material is doped with Eu^2+^, the Eu^2+^ ion plays the role of a luminescence center via its luminescent 4f^6^5d^1^ → 4f^7^ transition. Therefore, the 5d–4f transitions of Eu^2+^ in aluminate and silicate host materials generate similar broad emission spectra with a maximum in the blue-green region; the emission band is absent in systems without Eu^2+^ doping [57]. Moreover, the maximum peak positions are known to vary with the type of host material and are likely dependent on their surrounding configurations, such as symmetry, bond length, coordination, covalence, site size, and crystal field strength [57]. This probably occurs because of the displacement of the 5d energy level of Eu^2+^ in different crystal fields [56]. Therefore, numerous attempts have been made to tune the spectrum wavelength over a wide range by changing the composition and local crystal structure of the phosphors (Figure 1). For example, Eu^2+^ dopant ions in SrAl_2_O_4_ and Sr_4_Al_14_O_25_ are known to generate different emission wavelengths owing to their different crystalline structures (orthorhombic for Sr_4_Al_14_O_25_ and monoclinic for SrAl_2_O_4_) [44,58,59]. Kim et al. synthesized SrAl_2_O_4_:Eu^2+^,Dy^3+^ and Sr_4_Al_14_O_25_:Eu^2+^,Dy^3+^ as green and blue phosphors, respectively, by taking advantage of the different emission wavelengths [14]. 

Moreover, a new class of Eu^2+^-doped Sr_4_Al_2_O_7_ phosphors was recently reported to exhibit a longer wavelength relative to that of SrAl_2_O_4_:Eu^2+^ [31]. Zhang et al. also reported that the emission maximum shifted to a longer wavelength when the Al/Sr ratio increased in Eu^2+^-doped strontium aluminate phosphors [51]. Such spectral shifts in different host materials have been thoroughly explored with computational tools at the atomic and molecular levels using quantum mechanical methods, such as the density functional theory (DFT) and Hartree–Fock (HF) method. Theoretical investigations have indicated that the 4f–5d transition energy of lanthanides becomes redshifted after doping into the host lattice because it can affect the difference in energies of the lowest 4f^n^ and the first 4f^n−1^d levels in the lanthanide dopants [26]. Three important factors are thought to determine the spectroscopic redshift of lanthanide ions in host materials: centroid shift, crystal field splitting, and ligand polarization [23]. First, the centroid shift of the 5d orbital can be explained using the nephelauxetic effect. Given that the covalency between the luminescent center and its neighboring anions is proportional to the nephelauxetic effect, the centroid shift is expected to be in the F^−^ < Cl^−^ < Br^−^ < I^−^ < O^2−^ < S^2−^ order. Next, the type of the coordination polyhedron of anions around the luminescent center is known to strongly affect the highly susceptible nature of the 5d orbitals; this is referred to as crystal field splitting. Therefore, this effect is considered to play a crucial role in determining the spectral redshift of lanthanides in the host materials. Finally, ligand polarization was proposed as an important factor for determining the spectroscopic redshift [60]. The emission wavelengths of SrO:Eu^2+^ and Sr_2_SiO_4_:Eu^2+^ phosphors, which cannot be explained using the centroid shift, can be elucidated using ligand polarization.

### 2.2. Synthesis of Strontium Aluminate Phosphors

Various methods have been employed for the synthesis of lanthanide-doped strontium aluminate phosphors, such as sol–gel, solid-state reaction, combustion, microwave sintering, precursor, and coprecipitation. The selection of the appropriate synthesis method is crucial because it can significantly affect the quality of the luminescent material.

Among these methods, solid-state reactions have been extensively used for phosphor synthesis. This method is based on the chemical reactions between precursors in a powder form at high temperatures (1300–1600 °C). During this process, Eu^3+^ is reduced to Eu^2+^ in a reducing atmosphere; various reductants, such as H_2_ + N_2_, HI, and NH_4_I, are employed to facilitate this. The reductive atmosphere is crucial for avoiding sample decomposition or oxidization. Moreover, fluxing agents such as H_3_BO_3_ or LiF are often employed during this process to facilitate grain formation and crystal growth [62]. This method is popular in industrial settings because it is a conventional and robust method for the preparation of lanthanide-activated phosphors. For example, the solid-state reaction method was employed by Kim et al. and He et al. to synthesize SrAl_2_O_4_:Eu^2+^,Dy^3+^ and Eu^2+^-doped Sr_4_Al_2_O_7_ phosphors, respectively [12,48]. This method does not produce toxic or unwanted wastes and is, therefore, considered environmentally friendly. However, it is relatively difficult to accurately control and uniformly mix the individual components via chemical reactions in solid phases, in contrast to those in liquid phases. Additionally, the high-temperature sintering employed in this process for a relatively long reaction time often results in poor homogeneity in the product. Therefore, to avoid the formation of inhomogeneous grain boundaries, protocols involving pretreatment of the starting precursors at low temperatures (500–600 °C) followed by sintering at high temperatures (>1000 °C) have been employed [63,64].

The sol-gel method has been considered an attractive and straightforward alternative to the solid-state reaction method. In this process, solid particles suspended in a liquid (“sol”) are transformed into a three-dimensional network throughout the liquid (“gel”) via polycondensation reactions of molecular precursors. This process is conducted at a relatively low reaction temperature (~950 °C) to facilitate the uniform mixing of the starting materials and the formation of homogeneous products compared to those obtained from the solid-state reaction. This method has been employed for the synthesis of various mixed-metal oxides, nanomaterials, and organic–inorganic hybrids over the last few decades. Both nonaqueous and aqueous sol–gel methods have been employed. The aqueous sol–gel process is considered better than the nonaqueous process because the former facilitates the formation of a homogeneous solid-state structure at the atomic level, which is based on the chemical interactions among the precursor species in the mixture [65]. Misevicius et al. employed this concept in using the aqueous sol–gel approach for the synthesis of various Ce-doped strontium aluminates using glycolate intermediates, such as SrAl_2_O_4_, Sr_3_Al_2_O_6_, and Sr_4_Al_4_O_10_ [52].

Although the sol–gel technique can be successfully employed at a relatively low temperature (950 °C), both the solid-state reaction and sol–gel methods have unavoidable limitations, such as the extreme temperatures for long durations. To overcome them, the combustion method has been used as a promising technique for the synthesis of aluminate phosphors. This method is based on a self-sustaining exothermic redox reaction between the materials present in the starting mixture, which includes metal nitrates and urea as the oxidant and fuel, respectively. The reaction between the nitrates and fuel results in the formation of crystalline phases using the sufficient heat even at low temperatures. High levels of chemical homogeneity are also obtained because of their dissolution in the aqueous solution; this is followed by a uniform distribution of luminescent centers. In the Eu^2+^-doped phosphors, Eu^3+^ ions are reduced to Eu^2+^ by the gas released in this process [66]. This method has been extensively used to prepare various oxide materials, because it is relatively easy to avoid washing, filtration, and drying. This method is also preferred for the preparation of oxide materials at low temperatures. It is safe and efficient with respect to energy conservation because this process can be completed in only a few minutes (~5 min) at low temperatures. This reasoning was employed by Singh et al. to prepare the SrAl_12_O_19_:Eu^2+^ phosphor via the combustion method, which was found to conserve time, energy, and costs [49]. The combustion method has also been used to prepare dysprosium-doped strontium aluminate phosphors (SrAl_2_O_4_:Dy^3+^) and SrAl_2_O_4_:Eu^2+^,Nd^3+^ phosphors [27,42].

Finally, the precursor method involves the thermal decomposition of a single molecular precursor at high temperatures, which leads to the formation of nanophosphors. In this method, selection of the appropriate precursor is crucial for the synthesis of pure mixed oxides. Therefore, multimetallic complex compounds are typically preferred in this approach because they allow an intimate molecular-level contact of the metal ions. The selection of the appropriate complexation agent is also important for facilitating the production of complex compounds at low temperatures that can decompose easily. The molar ratios of the chemical elements in the final product can be easily controlled by changing the precursor concentration. This method is considered economically viable compared to other phosphor synthesis methods. The precursor method has also been used to prepare Tb^3+^-doped alkaline earth aluminates, such as SrAl_2_O_4_:Tb^3+^; scanning electron microscopy (SEM) analysis on these phosphors revealed that a homogeneous microstructure with a fine particle size was obtained [33].

In most of the aforementioned phosphor synthesis methods (solid-state reaction, sol–gel processing, combustion), crystalline materials with an average particle size of several tens of micrometers are produced. Therefore, to prepare nanometer-sized phosphors, post-treatment of the bulk phosphors that involves grinding of the large phosphor particles is necessary. Therefore, the particle size of the phosphor can be controlled. However, this process has several disadvantages, such as the unexpected oxidization of a few Eu^2+^ ions into inactive Eu^3+^ ions in the host lattice during the particle size reduction. In addition, the luminescent performance of the phosphors is degraded because of changes in the local coordination environment, such as crystallinity and lattice microstructure, around the dopants in the strontium aluminate hosts. For instance, Havasi et al. demonstrated the use of the ball-milling method for the production of submicrometer-sized particles of rare-earth-metal-doped strontium aluminate phosphors [50]. A comparative study of the mechanical stress resistance of various phosphors was used to observe the significant loss of long-persistent luminescence. However, this method is acceptable for use in industrial settings, although the elucidation of mechanisms involved and the feasibility of thermal restoration of the performance remain unclear. In addition to phosphor milling, various nanoengineering methods have been developed to synthesize ultrafine nanophosphors. For example, the use of surfactants or chelating reagents in hydro (solvo) thermal or microwave-assisted reactions was found to be effective for controlling the growth of the phosphor size [23]. In addition, laser ablation, template-directed synthesis, and microemulsion routes have been attempted to control the size and morphology of the phosphors [67].

### 2.3. Codoping of Strontium Aluminate Phosphors with Various Trivalent Lanthanide Ions

The luminescent properties of lanthanide-doped strontium aluminate phosphors can be further improved by codoping with other trivalent lanthanide ions as coactivators (such as Ln^3+^). The codoping of SrAl_2_O_4_:Eu^2+^ with Dy^3+^ resulted in a phosphor with an improved afterglow lifetime, intensity, and chemical stability compared to those of the SrAl_2_O_4_:Eu^2+^ phosphors [68]. In particular, the afterglow of SrAl_2_O_4_:Eu^2+^,Dy^3+^ was noted to last for over 10 h after exposure to illumination, and showcased a >10-fold increase in the initial intensity, making it an optimal material with persistent luminescence [43]. Interestingly, the position and shape of the luminescence emission band do not change, and the characteristic luminescence of Dy^3+^ is rarely observed in this coactivator-doped compound [56]. The direct excitation of Dy^3+^ or the energy transfer from Eu^2+^ to Dy^3+^ is noted to be negligible during the afterglow process. This implies that the luminescence center in these compounds does not change upon the incorporation of Dy^3+^, and it remains centered at Eu^2+^. Although the Dy^3+^ profiles are often observed only in the radioluminescence spectrum, they are likely to be hidden because of their weak intensities under the bright emission profiles of Eu^2+^ in most cases [41,69]. Other lanthanide ions such as Nd^3+^ and Tm^3+^ have also been employed as coactivators and were noted to exhibit similarly strong emission bands from the Eu^2+^ luminescence center without their characteristic photoluminescence [44,70].

The effect of coactivator concentration on the afterglow has also been investigated. Kim et al. reported that the SrAl_2_O_4_:Eu^2+^,Dy^3+^ phosphor exhibited the strongest persistent luminescence at a Dy^3+^/Eu^2+^ ratio of ~2.4 [12]; the afterglow luminance intensity was found to decrease as the Dy^3+^/Eu^2+^ ratio surpassed ~2.4, possibly because of the formation of the DyAlO_3_ by-product from residual and insoluble Dy^3+^ ions. The afterglow intensity from Eu^2+^ has been observed to increase as the dopant and codopant concentration increased [32]; the afterglow intensity was noted to decrease beyond its maximum value because of the concentration quenching effect, similar to that in the study discussed previously [12]. Such concentration effects involving the dopant and codopant can be explained by their distances.

Although codoping with lanthanide ions is known to significantly improve the luminescence, the exact role of Dy^3+^ in host materials remains unclear. The general understanding of codoping with lanthanide ions involves their contribution to the trapping centers of the material. For example, Dy^3+^ ions doped in the Sr^2+^ sites have been proposed to act as electron traps [38,70,71,72]. This is because the Dy^3+^ codopant ions are likely to replace the Sr^2+^ ions because of their similar radii and result in a +1 charge incompatibility. In addition, the concentration ratio of Eu^2+^ and Eu^3+^ after the chemical reduction process can be modified via the codopant ions because they can stabilize the Eu valences in the phosphors [73]. Similarly, Dy^3+^ ions are expected to modify the environment of Eu^2+^ ions in the SrAl_2_O_4_ host materials. Other possibilities include the trapping of a hole by Dy^3+^ or the attraction of other defects for charge compensation [56].

### 2.4. Mechanisms of Long-Persistent Luminescence from Lanthanide-Doped Strontium Aluminate Phosphors

**Figure 2:**
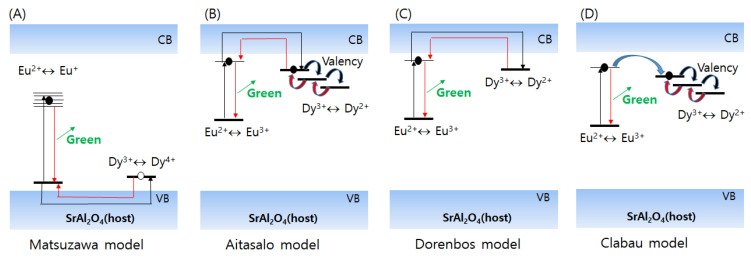
The (**A**) Matsuzawa, (**B**) Aitasalo, (**C**) Dorenbos, (**D**) Clabau models for the photoluminescence of SrAl_2_O_4_:Eu^2+^,Dy^3+^.

Several models have been suggested to elucidate the persistent luminescence of codoped SrAl_2_O_4_:Eu^2+^,Dy^3+^ (Figure 2). The differences in these models arise from the assumptions regarding the charge carriers (holes or electrons), pathways of the charge carriers (valence band, conduction band, trapping centers), nature of trapping centers (intrinsic defects or codopants), and excitation mechanism of luminescence. Although the elucidation of persistent luminescence mechanisms in codoped SrAl_2_O_4_:Eu^2+^,Dy^3+^ remains incomplete, the general background in all these models involves the generation of migrating charge carriers during excitation and subsequent localization in trapping centers. The important models that try to explain the mechanism behind the long afterglow in the codoped SrAl_2_O_4_:Eu^2+^,Dy^3+^ phosphor are discussed in this section.

The first of these models was proposed by Matsuzawa et al. [5]. Briefly, the model involves the generation of a hole during Eu^2+^ excitation and subsequent Eu^+^ formation, subsequent release and migration of the hole to Eu^+^ through the valence band at a high temperature, and generation of Eu^2+^ in the excited state; a photon is also subsequently released from the excited Eu^2+^. In this model, Dy acts as a hole-trapping center by localizing the holes released from Eu^2+^, and it facilitates the conversion of Dy^3+^ to Dy^4+^. Therefore, the hole release and migration from the trapping centers to Eu^+^ is crucial to determining the afterglow. However, the main problem in this model involves the energetically unfavorable formation of Eu^+^.

To overcome this limitation, Aitasalo et al. proposed a new model for codoped SrAl_2_O_4_:Eu^2+^,Dy^3+^ [43,74,75]. In this model, the possibility of Eu^+^ formation is excluded, and the formation of electron traps and migration of electrons is considered instead of hole traps. Trivalent rare-earth codopants play an important role in trapping electrons at the defect level in this model. As shown in Figure 2B, electrons from the Eu^2+^ excited state are thermally promoted to the conduction band and are eventually trapped on defect levels, such as oxygen vacancies, trivalent rare-earth ions, cation vacancies, and interstitial ions. The charge carriers subsequently migrate back to the luminescence center, Eu^2+^, which is followed by the characteristic luminescence of the transition of Eu^2+^ to the ground state. Studies involving X-ray absorption near-edge structure (XANES) and extended X-ray absorption fine structure (EXAFS) analyses have verified this model by confirming the accumulation of Eu^3+^ during excitation. The Eu^2+^/Eu^3+^ oxidation has also been observed in XANES measurements of SrAl_2_O_4_:Eu^2+^,Dy^3+^, which support this model [46]. Qiu et al. suggested that such trapping and detrapping processes from the defect levels can be repetitive [38].

Dorenbos et al. proposed a similar model [72], in which the dopant and codopant energy levels in the bandgap of the SrAl_2_O_4_ host material were estimated. In particular, the energy levels of Eu^3+^ and Dy^3+^ were presumed to be positioned immediately below the bottom of the conduction band, and ~0.9 eV below the bottom of the conduction band, respectively. Because the difference between these energy levels and that of the conduction band is small, thermal ionization is likely to occur at room temperature. The electron that migrates through the conduction band is eventually trapped by Dy^3+^, which is followed by recharging to Dy^2+^. Subsequently, the electron can be thermally released from Dy^2+^ and migrates to the excited Eu^2+^ center, which eventually leads to the photon emission. The main difference between this model and the Aitasalo model is the type of electron trap discussed. The model presented by Dorenbos et al. only considers trivalent rare-earth ions, whereas that by Aitasalo et al. suggests various kinds of defects, such as oxygen vacancies and trivalent rare-earth ions [43,74,75].

Clabau et al. proposed a new model that involved the formation of Eu^3+^ and electron migration, in a manner similar to that discussed in the abovementioned models [54]. Based on results from EPR experiments, the ionization of Eu^2+^ to Eu^3+^ was assumed to occur after excitation by UV irradiation. The main difference between this and the previous models involves the direct migration of electrons between the luminescence and trapping centers and not via the conduction band. This can occur if the energy levels of Eu^3+^ and trapping centers are located close to each other.

Broadly speaking, all the aforementioned models describe the generation of charge carriers during excitation, and their subsequent localization in trapping centers postmigration. The charge carrier is subsequently released from the trapping centers and eventually recombined with the excited luminescent center. The model that best represents the persistent luminescence in SrAl_2_O_4_:Eu^2+^,Dy^3+^ is likely to involve the creation of Eu^3+^ and the radiative Eu^2+^ transition to the ground state. Moreover, there may be several trapping centers present in the phosphor, although their exact nature remains unclear.

The use of multiple codopants has also been attempted because of the effectiveness of codoping for the enhancement of the luminescence of Eu^2+^. Song et al. and Li et al. employed Tb^3+^ and Er^3+^ as additional codopants in Sr_4_Al_14_O_25_:Eu^2+^,Dy^3^, respectively [45,47]. In addition, Havasi et al. demonstrated the photoluminescence properties of Sr_4_Al_14_O_25_:Eu^2+^,Dy^3^,Ho^3+^, which was synthesized using Dy^3+^ and Ho^3+^ as additional codopants [50]. During sample preparation in this study, Dy^3+^ in Sr_4_Al_14_O_25_:Eu^2+^/Dy^3+^ was presumed to be partially substituted with Ho^3+^, which remarkably increased the photoluminescence intensity and shifted the main emission peaks. This was explained via the effect of Dy^3+^/Ho^3+^ on the trapping/de-trapping and energy transfer processes in this phosphor [76].

## 3. Calcium Aluminate Phosphors

Calcium aluminate is also considered as a useful host matrix material for phosphors (Table 2). Similar to strontium aluminate, calcium aluminate has been known to exhibit a bright emission over a wide visible range and a high energy efficiency, quenching temperature, and chemical stability. In addition, its high toughness, strength, and high-temperature resistance facilitate its use as cement materials [77]. In particular, calcium aluminates have been applied as dental cements and bone grafts because of their bioactive, biocompatible, physical, and mechanical properties.

### 3.1. Calcium Aluminate Phosphors with Diverse Colors

**Figure 3:**
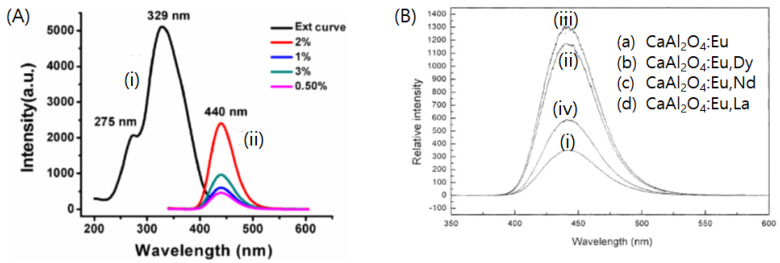
Photoluminescence spectra for lanthanide doped calcium aluminate phosphors (**A**) Photoluminescence excitation (i) and emission (ii) spectra for CaAl_2_O_4_:Eu^2+^. Adapted from Ref. [30] under Creative Commons Attribution (CC BY) license. (**B**) Emission spectra of (i) CaAl_2_O_4_:Eu^2+^, (ii) CaAl_2_O_4_:Eu^2+^,Dy^3+^, (iii) CaAl_2_O_4_:Eu^2+^,Nd^3+^, (iv) CaAl_2_O_4_:Eu^2+^,La^3+^. Adapted with permission from [82]. Copyright Elsevier, 2003.

Calcium aluminates belong to the spinel group of minerals, similar to the other alkaline earth aluminates. The typical chemical representation of calcium aluminate is CaAl_2_O_4_, and it exists in the monoclinic or orthorhombic forms [83]. As discussed earlier, the host crystal structure and activator are the important factors for determining the main emission peaks of aluminate-based phosphors. For example, CaAl_2_O_4_:Eu^2+^ is known to exhibit blue light emission, which corresponds to a shorter emission wavelength in the SrAl_2_O_4_:Eu^2+^ system. Among the various lanthanide-doped calcium aluminate phosphors, CaAl_2_O_4_:Eu^2+^ is extensively used as a phosphor material (Figure 3). As an emission center, Eu^2+^ is known to emit blue light via the 4f^6^5d → 4f^7^ transition, whose peak is located at ~442 nm [78]. Its absorption is observed at near-UV light, which is similar to that via LED chips. Although there are fewer studies on CaAl_2_O_4_ phosphors than those on SrAl_2_O_4_, the former has attracted considerable recent interest as a luminescent host owing to its high color purity and stability; it also meets the high-efficiency-based requirements for novel blue-emitting phosphors [84].

Several activators have been experimented as dopants for CaAl_2_O_4_. For example, Tb^3+^, which has been doped into CaAl_2_O_4_ in several studies, can substitute a Ca^2+^ ion as Eu^2+^, which is followed by the formation of a charge defect [85]; CaAl_2_O_4_:Tb^3+^ was found to exhibit an emission wavelength of 545 nm, which corresponded to the f-f transitions of Tb^3+^. La^3+^-doped CaAl_2_O_4_ synthesized via the sol–gel method exhibited an emission peak at 395 nm in the blue region [79]; its emission band intensity was observed to be stronger than that of CaAl_2_O_4_:Tb^3+^. The particle sizes of CaAl_2_O_4_:La^3+^ and CaAl_2_O_4_:Tb^3+^ were obtained as 27 nm and 31 nm, respectively, and both had no effect on the phase composition of CaAl_2_O_4_ [79]. Additionally, several studies have attempted the Ce^3+^ ion doping of CaAl_2_O_4_ and have resulted in a much shorter emission wavelength (330–350 nm). This emission band corresponds to the 5d–4f transitions of Ce^3+^ ions. The broad emission band was found to be remarkably intense because of the transition being parity-allowed [30]. Nonlanthanide ions, such as Mn, have also been employed as activators in CaAl_2_O_4_. Mn-doped aluminate phosphors synthesized via the combustion process were found to exhibit red emission from the Mn^4+^ ions [86]; Mn ions were noted to exist in the CaAl_2_O_4_ host material in both Mn^2+^ and Mn^4+^ states; they also occupied distorted lattice sites in the host matrix.

### 3.2. Codoping of Calcium Aluminate Phosphors with Various Trivalent Lanthanide Ions

Similar to strontium aluminate phosphors, codoping of CaAl_2_O_4_ phosphor materials with various trivalent lanthanide ions has been attempted to improve their luminescence properties. Lin et al. suggested that the incorporation of Dy^3+^, Nd^3+^, and La^3+^ can possibly enhance the brightness and persistent afterglow time [82]. Regardless of the type of codopant ions used, the excitation and emission of these three phosphors resulted in spectra that were similar in shape to those of the CaAl_2_O_4_:Eu^2+^ phosphor, which corresponded to the 4f^7^–4f^6^5d inter-configuration transitions of Eu^2+^ ions. Among these phosphors, the afterglow from CaAl_2_O_4_:Eu^2+^,Nd^3+^ was found to be the brightest and with the longest duration.

The CaAl_2_O_4_:Eu^2+^,Nd^3+^ phosphor has, therefore, been extensively investigated. Zhao et al. investigated the UV-excited luminescence of CaAl_2_O_4_:Eu^2+^,Nd^3+^ and observed a broad band in the blue region (λ_max_ = 440 nm) that arose from the 5d–4f transitions of Eu^2+^ [80]. The bright afterglow luminescence was observed for a long duration. This long-persistent luminescence was presumed to result from the trapping–transporting–detrapping of the holes, a process in which Nd^3+^ ions behaved as hole traps between the ground and the excited states of the Eu^2+^ ion. Kim et al. reported the optimized composition of the Eu^2+^ activator and Nd^3+^ coactivator for CaAl_2_O_4_ [81]. The introduction of Nd^3+^ into the CaAl_2_O_4_:Eu^2+^ system was found to significantly boost its phosphorescence intensity and lifetime, similar to that of Dy^3+^ doping in the SrAl_2_O_4_:Eu^2+^ system. Composition-based studies on the activator (Eu^2+^) and coactivator (Nd^3+^) have revealed that the afterglow intensity and lifetime were strongly affected by the concentrations of Eu^2+^ and Nd^3+^ in CaAl_2_O_4_:Eu^2+^,Nd^3+^; this implies that the optimization of the activator and coactivator concentrations appears to be important for obtaining a high intensity of phosphorescence. The afterglow of phosphors with various concentrations of the activator (Eu^2+^) and coactivator (Nd^3+^) were measured, and ~0.006 mol of Eu^2+^ per mol of CaAl_2_O_4_:Eu^2+^,Nd^3+^, and an Nd^3+^/Eu^2+^ ratio of 1 were found to result in a product with the brightest phosphorescence emission for the longest duration. This optimized concentration was noted to be much lower than that of Eu^2+^ (~0.935 mol per mol of SrAl_2_O_4_:Eu^2+^,Dy^3+^) and Dy^3+^ (~2.244 mol per mol of SrAl_2_O_4_:Eu^2+^,Dy^3+^) in the green-emitting SrAl_2_O_4_:Eu^2+^,Dy^3+^ phosphor, probably because of their relatively large sizes.

### 3.3. Synthesis of Calcium Aluminate Phosphors

The CaAl_2_O_4_:Eu^2+^ phosphors are typically prepared in a manner similar to that for the SrAl_2_O_4_:Eu^2+^ phosphors. The high-temperature solid-state reaction is intensively used for the preparation of CaAl_2_O_4_:Eu^2+^; however, the high calcination temperature and the formation of heterogeneous particles with relatively large microscale sizes limit their applications. Therefore, the various properties of the fluxing agent in the solid-state process have been modified to control the particle size and reduce the sintering temperature. This is attempted because the fluxing agent is presumed to facilitate the incorporation of lanthanide ions in the matrix lattice. For example, the effect of the concentration of H_3_BO_3_ as a fluxing agent on the structure, morphology, and luminescent properties of Ca_1−x_Al_2_O_4_:xEu^2+^ have been explored by Zeng et al. [78] A comparison of different amounts of H_3_BO_3_ for phosphor synthesis revealed that an H_3_BO_3_ mass ratio of 0.5 wt.% resulted in a product with an adequate morphology without agglomeration and the best luminous intensity among the various samples. Kim et al. also presented a comparative study of varying amounts of H_3_BO_3_ [81], which suggested that 0.25 mol per mol of CaAl_2_O_4_:Eu^2+^,Nd^3+^ resulted in the brightest phosphorescence among the various samples; however, a higher concentration of H_3_BO_3_ was found to produce a hardened final product, which can create difficulties in the subsequent mortar grounding process.

Several alternative methods involving liquid phases, such as sol–gel, combustion, coprecipitation, and microwaves, have been employed for the preparation of CaAl_2_O_4_:Eu^2^+, in a manner similar to that for strontium aluminate phosphors. In the liquid phase, each component can be uniformly mixed and accurately controlled. For example, the sol–gel process facilitates the homogeneous mixing of the starting materials and synthesis at a relatively low reaction temperature, which results in the formation of homogeneous products with a fine grain size [37,82]. The combustion method is another efficient technique for the preparation of CaAl_2_O_4_:Eu^2+^ at a relatively low temperature and is known for being facile, safe, quick, cost-effective, and energy conserving. Zhao et al. reported that the CaAl_2_O_4_ phase was formed at a combustion initiation temperature of 400 °C via the combustion method; this method resulted in the formation of persistent luminescent CaAl_2_O_4_:Eu^2+^-based phosphors with bright phosphorescence and a long duration [80].

Several methods have been recently attempted for the preparation of Eu^2+^-doped calcium aluminate phosphors. Jia et al. employed a LASER-heated pedestal growth method to synthesize the CaAl_2_O_4_:Eu^2+^,Nd^3+^ phosphor, which resulted in bright and long-persistent phosphorescence being observed from the final product [35]. Katsumata et al. attempted a loading zone technique to grow a single-crystal CaAl_2_O_4_:Eu^2+^,Nd^3+^ phosphor, and the resulting product exhibited luminescent properties similar to those of the previously reported SrAl_2_O_4_:Eu^2+^,Dy^3+^ phosphor [34].

## 4. Barium Aluminates

Barium aluminate phosphors are persistent and exhibit high luminescent intensity, long afterglow time, and chemical stability (Table 3). Moreover, their synthesis does not require a reducing atmosphere for dopant reduction [87].

Barium aluminate (BaAl_2_O_4_) has a stuffed tridymite structure that is derived from the SiO_2_ β-tridymite structure observed in other alkaline earth aluminates [96]. It has a hexagonal phase, which is different from those of strontium aluminate and calcium aluminate. Barium aluminates exhibit a stable monoclinic phase at low temperatures which are transformed from the hexagonal phase after cooling. Barium aluminate (BaAl_2_O_4_) also has a high melting point (1815 °C), and exhibits adequate chemical stability and decent dielectric, pyroelectric, and hydraulic-hardening properties [97]. In its structure, two different sites are available for Ba^2+^: one with a C_3_ symmetry and a relatively longer Ba–O distance (2.86–2.87 Å), and the other with a C_1_ symmetry and a relatively shorter Ba–O distance (2.69 Å) [94]. Ba^2+^ has a larger ionic radius (1.34 A°) than that of most other rare-earth ions; this facilitates the straightforward substitution of vacant Ba^2+^ sites with lanthanide ions upon doping [88].

### 4.1. Synthesis of Barium Aluminate Phosphors

Various techniques have been employed to prepare barium aluminate phosphors in a manner similar to those for strontium and calcium aluminate phosphors, such as solid-state reactions, combustion methods, and microwave heating techniques.

The conventional solid-state reaction method has been typically used to successfully prepare barium aluminate phosphors [98]. In contrast to the synthesis of strontium and calcium aluminates, barium aluminate can be synthesized in an oxidizing atmosphere. Peng et al. were the first to report the reduction ofEu^3+^ to Eu^2+^ in an oxidizing atmosphere (air) via a high-temperature solid-state reaction for doping into an AlO_4_ tetrahedron in the BaAl_2_O_4_ crystal [8]. The tetrahedral AlO_4_ anion groups can form a hard three-dimensional network, which can induce the Eu^3+^ reduction even when the barium aluminate phosphors are prepared in air. Therefore, diverse atmospheric conditions such as reducing, weak reducing, and oxidizing atmospheres have been employed to synthesize barium aluminate phosphors. This method is straightforward and does not require expensive or sophisticated equipment; it is also convenient for large-scale industrial production. In addition, it can produce a structurally pure final product with desirable properties, depending on the final sintering temperatures.

Combustion synthesis is another extensively used method for the preparation of barium aluminate phosphors. Annah et al. prepared trivalent lanthanide codoped BaAl_2_O_4_:Eu^2+^ phosphors via the combustion method at an initiating temperature of 600 °C and annealing at 1000 °C [95]. The annealing process was found to have no effect on the general properties of the phosphors. Rodrigues et al. also employed this method at similarly low temperatures between 400 and 600 °C; Mothudi et al. synthesized BaAl_2_O_4_:Eu^2+^,Dy^3+^ at a combustion initiation temperature of 500 °C with urea as an organic fuel for combustion [39,99]. The combustion method can be performed at a much lower temperature compared to that in the solid-state reaction, and it is considered to be a simple, time-saving, and cost-effective technique. As previously discussed, it can produce smaller crystals than those obtained from a solid-state reaction. The size of particles produced via the combustion method was found to decrease from 98 nm to 85 nm as the reaction temperature increased from 400 to 600 °C [96]. Another difference between these two methods involves the number of traps produced; one trap is formed in the combustion method, whereas three traps are formed in the solid-state method, which hints at the formation of various defect structures in the materials, depending on the synthesis methods.

BaAl_2_O_4_ phosphors have also been synthesized by microwave heating. Zhang et al. successfully synthesized BaAl_2_O_4_ using BaCO_3_ and Al(OH)_3_ powders as raw materials via microwave sintering [100]. These phosphors were characterized by thermogravimetry–differential scanning calorimetry (TG–DSC), X-ray diffraction (XRD), and optical microscopy; this method was found to be feasible for the preparation of persistent luminescence materials of barium aluminates. Although this technique is not employed as frequently as the two previously discussed methods, it has several advantages, such as the low temperatures and short durations in sintering, a simple and easy setup, cost-effective energy source, and high rate of synthesis.

### 4.2. Barium Aluminate Phosphors with Various Colors

**Figure 4:**
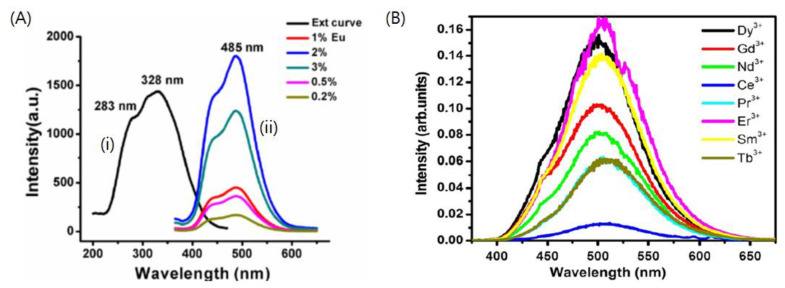
Photoluminescence spectra for lanthanide doped barium aluminate phosphors (**A**) Photoluminescence excitation (i) and emission (ii) spectra for BaAl_2_O_4_:Eu^2+^. Adapted from [30] under Creative Commons Attribution (CC BY) license. (**B**) Photoluminescence emission spectra of BaAl_2_O_4_:Eu^2+^, Re^3+^ (Re = Dy^3+^, Er^3+^, Sm^3+^, Gd^3+^, Ce^3+^, Pr^3+^ and Nd^3+^). Adapted with permissions from [95]. Copyright Elsevier, 2012.

Eu^2+^ is the most popular rare-earth element for doping in BaAl_2_O_4_, similar to that in the alkali metal-based aluminate phosphors [98] (Figure 4). BaAl_2_O_4_ phosphors have been considered for application in plasma display panels (PDPs) and mechanoluminescence (ML) dosimetry owing to their enhanced luminescence intensity, long-lasting duration, and suitable emitting colors via Eu^2+^ doping. The peak of the broad excitation spectra is observed at 340 nm, and the emission spectra are present in the blue–green region under vacuum/ultraviolet (VUV) light excitation, which corresponds to the 5d–4f transition of Eu^2+^. The spectral peak does not appear uniform, which implies the occurrence of multiple events at the luminescent centers. Peng et al. reported that Eu^2+^ ions can occupy 2 different lattice sites after doping in BaAl_2_O_4_: the Eu^2+^ ion in the first site exhibited a major emission peak at 495 nm, and the Eu^2+^ ion in the other exhibited a weak emission peak at 530 nm [8]. The main emission peak is noted to be positioned between the emission peaks of SrAl_2_O_4_:Eu^2+^ (528 nm) and CaAl_2_O_4_:Eu^2+^ (449 nm), implying that the crystal structure of the host plays a crucial role in determining the main emission peaks of aluminate-based phosphors. In addition, Stefani et al. observed that the relative intensity of the two emission peaks in BaAl_2_O_4_:Eu^2+^ can be modified by varying the dopant and codopant concentrations [87]. The intensity of the emission peak at a shorter wavelength was observed to increase as the dopant and codopant concentrations increased, which suggested that Eu^2+^ preferentially occupied the Ba^2+^ site responsible for longer wavelength emission and subsequently occupied another site corresponding to the shorter wavelength emission. Feilong et al. reported 1 mol.% as the optimal Eu^2+^ concentration for the enhancement of luminescent intensity of BaAl_2_O_4_ [91]. Roh et al. also studied the effect of Eu^2+^ concentration on the photoluminescence of these phosphors [92]. The photoluminescence efficiency was noted to increase as the Eu^2+^ concentration increased up to 3 mol.%. and concentrations greater than 3 mol.% quenched the photoluminescence of BaAl_2_O_4_:Eu^2+^.

In addition to Eu^2+^ ions, Cr^3+^ ions have also been employed as dopants in BaAl_2_O_4_. Singh et al. prepared red-emitting BaAl_2_O_4_:Cr^3+^ phosphors via the urea combustion method [89]. The excitation spectra of this synthesized phosphor featured two broad bands with high intensities at 421 and 552 nm, which were ascribed to the Cr^3+^ ions in octahedral symmetry. The emission peak observed at 750 nm corresponded to the transition from Cr^3+^ ions. Vrankic et al. investigated the oxidation state of Cr dopant in a Cr-doped BaAl_2_O_4_ structure using XRD and synchrotron-based X-ray absorption spectroscopy (XAS) [90]. Two different oxidation states for chromium ions were found. Cr^6+^ was observed in a small amount in an impure phase (BaCrO_4_), whereas Cr^3+^ was noted to participate in the formation of the doped BaAl_2_O_4_:Cr^3+^ phase, in which it behaved as a defect.

### 4.3. Codoping of Barium Aluminate Phosphors with Various Trivalent Lanthanide Ions

The persistent luminescence of lanthanide-doped BaAl_2_O_4_ phosphors can also be enhanced by codoping with trivalent lanthanide ions, similar to that in the other alkaline earth aluminates. Among the various lanthanide elements that have been used as codopants, Eu^2+^ and Dy^3+^ have been particularly successful as codopants in the synthesis of polycrystalline barium aluminate phosphors (BaAl_2_O_4_:Eu^2+^,Dy^3+^) [36,87,93,99]. The photoluminescence efficiency of the Eu^2+^-doped BaAl_2_O_4_ phosphor was observed to increase after codoping with Dy^3+^; this phosphor exhibited afterglow properties with the longest duration among the various MAl_2_O_4_:Eu^2+^,Dy^3+^ phosphors (M = Sr, Ca, Ba).

Liu et al. reported that the codoping of BaAl_2_O_4_:Eu^2+^ with Dy^3+^ did not modify the positions of either the emission band or the excitation band; however the luminescence intensity and afterglow duration of the phosphor increased [36]. The generally accepted mechanism for the photoluminescence of BaAl_2_O_4_:Eu^2+^,Dy^3+^ is similar to that ofSrAl_2_O_4_:Eu^2+^,Dy^3+^ and includes the following steps: (1) electron migration induced by UV radiation from the 4f^6^5d^1^ levels in Eu^2+^ to the conduction band, (2) electron trapping from the conduction band to defects such as oxygen vacancies or codopant ions, (3) reverse electron migration from traps to the 4f^6^5d^1^ levels in Eu^2+^, and (4) radiative relaxation of the returned electron to the ground state of the luminescent center, Eu^2+^. In this process, the type of codopant can affect the formation of electron traps, and therefore, the Dy^3+^ trap levels are presumed to be responsible for the long afterglow phosphorescence [36,93]. The effect of Dy^3+^ concentration on the afterglow of BaAl_2_O_4_:Eu^2+^,Dy^3+^ has also been investigated [87,99]. Stefani et al. found that the concentrations of both Eu^2+^ and Dy^3+^ play an important role in the determination of the crystallinity and luminescence properties of BaAl_2_O_4_:Eu^2+^,Dy^3+^ phosphors [87]. Rodrigues et al. investigated the concentration of Eu^2+^/Dy^3+^ (in mol.% with respect to the amount of Ba) from 0.1/0.1 to 1.0/3.0, and found that the luminescence intensity increased as the Eu^2+^ and Dy^3+^ concentrations increased [99]. The high concentration of Eu^2+^ and the resulting enhancement of luminescence can be explained by an increase in the number of emitting centers. Increased Dy^3+^ concentration is also presumed to reduce the distance between the electron traps and the emitting center, which increases the efficiency of energy transfer.

Cr^3+^ ions have also been employed as codopants in BaAl_2_O_4_:Eu^2+^. Ryu et al. synthesized BaAl_2_O_4_:Eu^2+^,Cr^3+^ using various concentrations of Cr^3+^ [94]. Crystalline fibers were obtained via the different concentrations of Cr^3+^ (0.01, 0.05, and 0.1 mol%); fibers with larger dimensions were obtained at higher concentrations of doped Cr.

Ho^3+^ ions have also been codoped in BaAl_2_O_4_:Eu^2+^ to facilitate the enhancement of photoluminescence. Liu et al. reported that the BaAl_2_O_4_:Eu^2+^,Ho^3+^ samples exhibited excitation and emission spectra with shapes and positions similar to those from BaAl_2_O_4_:Eu^2+^,Dy^3+^ but with slightly lower emission intensities [101].

Annah et al. systematically investigated the use of several trivalent rare-earth ions (Dy^3+^, Nd^3+^, Gd^3+^, Sm^3+^, Ce^3+^, Er^3+^, Pr^3+^, and Tb^3+^) as codopants in BaAl_2_O_4_:Eu^2+^ [95]. All tested samples were found to exhibit blue–green emission at ~500nm, which corresponds to the 4f^6^d^1^–4f^7^ transitions of Eu^2+^; no changes were observed in the phase structure after codoping. The emission intensity of the Er^3+^-codoped phosphor was noted to be the highest, and the afterglow of the Nd^3+^-codoped phosphor was observed to be the longest.

## 5. Outlook

**Figure 5:**
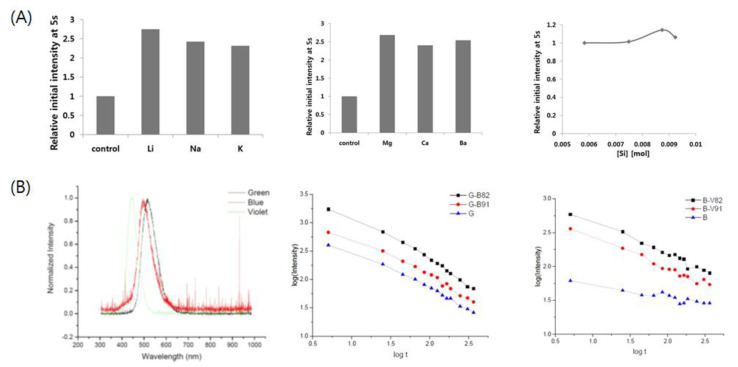
Various efforts to enhance the photoluminescence intensity of lanthanide-doped alkaline earth aluminate phosphors (**A**) Enhanced photoluminescence intensity by alkali metal (left), alkaline earth metal (middle), and Si (right) ions doping. Adapted from [12] under Creative Commons Attribution (CC BY) license. (**B**) (left) Emission spectra of the green (SrAl_2_O_4_:Eu^2+^,Dy^3+^), blue (Sr_4_Al_14_O_25_:Eu^2+^,Dy^3+^), and violet (CaAl_2_O_4_:Eu^2+^,Nd^3+^) phosphors used for the phosphor mixing method with the aim of energy transfer between them. (middle) Photoluminescence decay curves in log scale for the green and blue phosphors mixed sample, showing enhanced luminescence intensity of green phosphor by mixing with the blue phosphor. (right) Photoluminescence decay curves in log scale for the blue and violet phosphors mixed sample, showing enhanced luminescence intensity of blue phosphor by mixing with the violet phosphor. Adapted from [14] under Creative Commons Attribution (CC BY) license.

Lanthanide-activated alkaline earth aluminates phosphors are excellent luminescent materials and can have extensive applications. In this review, lanthanide-doped strontium aluminate-, calcium aluminate-, and barium-aluminate-based phosphors, which are among the popular alkaline earth aluminates, were discussed with an emphasis on their synthesis methods, phosphorescence mechanisms, and the effects of dopants and codopants on phosphor properties. This systematic review featuring the development of various lanthanide-activated alkaline earth aluminate phosphors is expected to stimulate further research on lanthanide-based phosphors for applications in a wide range of areas. Various attempts have been made to optimize the synthesis methods and compositions of lanthanide-activated alkaline earth aluminates phosphors to improve their long-persistent luminescence properties; however, recent strategies involving nanomaterial engineering, which have been successfully implemented for different types of nanoparticles, can also be adopted to further enhance the luminescence properties of phosphors (Figure 5).

Energy transfer between the different lanthanide ions in dual-emitting lanthanide-ion-codoped phosphors has been utilized to facilitate ratiometric temperature sensing and enhancement of photoluminescence of the phosphors. Energy transfer between two lanthanide codoped ions has been observed in several dual-color emitting phosphors, such as Ba_2_Y(BO_3_)_2_Cl:Bi^3+^,Eu^3+^, BaLu_6_(Si_2_O_7_)_2_(Si_3_O_10_):Ce^3+^,Tb^3+^, and LaOBr:Ce^3+^,Tb^3+^ [47,102]. Two emission peaks were observed herein because of the presence of two luminescence centers in these dual-emitting phosphors; their ratio was found to change with the extent of energy transfer. Applying this phenomenon, Zhang et al. demonstrated the ratiometric temperature sensing of LaOBr:Ce^3+^,Tb^3+^ over a wide temperature-sensing range (293–443 K) with a sensitivity of 0.42% K^−1^ [103]. This approach has also been recently demonstrated in lanthanide-codoped aluminate phosphors with various colors, such as SrAl_2_O_4_:Eu^2+^,Dy^3+^, Sr_4_Al_14_O_25_:Eu^2+^,Dy^3+^, and CaAl_2_O_4_:Eu^2+^,Nd^3+^ as green, blue, and violet phosphors, respectively [14]. Novel protocols have been developed based on the effect of energy transfer between spectrally different phosphors, and they were noted to significantly improve the afterglow intensities and lifetimes of green and blue phosphors. Multistep energy transfer between the three phosphors with different colors was also achieved, and a much higher afterglow intensity was generated: ∼2 times that via single-step energy transfer. Therefore, it is vital to consider the synthesis of various lanthanide-doped alkaline earth aluminate phosphors as a new strategy for the development of bright and long-persistent phosphors.

Distortion of crystal fields has also been induced via the introduction of impurities to facilitate the tuning of the luminescence properties of phosphor crystals [104]. Various alkali metals have been employed as dopants to increase the afterglow intensity and lifetime. Dhananjaya et al. observed that the photoluminescence intensity of the Gd_2_O_3_:Eu^3+^ phosphor remarkably increased after the incorporation of Li+, Na+, and K+ into this phosphor [105]. Kim et al. also reported that the doping of SrAl_2_O_4_:Eu^2+^,Dy^3+^, and CaAl_2_O_4_:Eu^2+^,Nd^3+^ phosphor with alkali metals (Li^+^, Na^+^, K^+^) and alkaline earth metals (Mg^2+^, Ca^2+^, Ba^2+^) can significantly boost the phosphorescence intensity and increase the afterglow lifetime [12,81]. These apparently imply that Si^4+^ doping is also effective for enhancing the phosphorescence intensity. Upon the incorporation of Si^4+^ into the SrAl_2_O_4_ crystal, the local symmetry of the crystal structure was presumed to be broken owing to the smaller size of Si^4+^ (~40 pm) compared to that of Al^3+^ (53 pm). Therefore, an increase in luminescence was observed for both phosphors at the optimal concentration of Si^4+^. The effect of incorporation of additional impurities on photoluminescence is presumed to be a result of the local distortion of the crystal field surrounding the luminescence center, which is known to considerably affect the f–d transitions. Such impurity effects are expected to play an important role in the future development of effective codopants.

Ideas for the straightforward fabrication of nanoparticles with desirable shapes and sizes have also been suggested. Liu et al. attempted to control the size, shape, and surface properties of rare-earth-doped nanomaterials at the atomic scale using oleate anions (OA^−^) and molecules (OAH) [106]. This level of control was observed to facilitate the fabrication of various sub-50 nm-sized monodispersed nanoparticles. Similarly, Sui et al. reported the use of oleate salts as ligands that can shorten the reaction time (down to 5 min) during the synthesis of the ultrasmall (~13 nm) hexagonal phase of the NaYF_3_ nanocrystals; this was facilitated via induction of the orderly arrangement of Y^3+^ and lowering of the energy barrier for the phase transition to occur [107].

Overall, recent approaches based on nanomaterial engineering can be expected to be readily expanded to lanthanide-doped alkaline earth aluminate phosphor systems for the enhancement of their photoluminescence properties. However, results from previous studies on the design of phosphors for obtaining desirable sizes of the phosphor particles need to be carefully considered. Most of the studies discussed in this review involve bulk structures; however, the properties of nanoparticles, such as luminescence and physical and chemical properties, can be different at the nanoscale. The coordination complex of a doped lanthanide ion can be easily distorted in nanoparticles compared to that in the bulk lattice because nanophosphors typically have large surface areas and high densities of interfacial boundaries. Recent approaches based on theoretical modeling are expected to play an essential role in the estimation of the photoluminescence characteristics and mechanisms in newly designed nanophosphors. The development of new lanthanide-doped phosphors should include systematic characterization to investigate the sizes and scales involved.

## 6. Conclusions

Considerable attempts have been made to develop bright and long-persistent lanthanide-doped alkaline earth aluminate phosphors for replacing the conventionally and extensively used ZnS phosphors; as a result, the luminescence properties of such phosphors have been significantly improved. Various synthesis methods have been employed to prepare lanthanide-doped alkaline earth aluminate phosphors, and their pros and cons have been found to arise from the different reaction temperatures, reaction phases (solid or liquid), and particle size of the product. The luminescence properties of these aluminate phosphors are mainly determined by the compositions of the doped lanthanide ions and the host matrix. The localized environment surrounding the lanthanide activator, which is a luminescence center, appears to play a crucial role in persistent luminescence. It is important to determine the effect of the synthesis steps and compositions of phosphors on the persistent luminescence because subtle changes in the phosphor synthesis conditions can lead to significant variations in their luminescence properties. The development of lanthanide-doped alkaline earth aluminate phosphors has great potential; such phosphors can also be expected to find application in a wide range of areas.

## Figures and Tables

**Table 1:** Comparison of reported studies for the synthesis of lanthanide-doped strontium aluminate phosphors.

Host Material	Activator	Co-Activator	Synthesis Method	Color or λ	Remarks	Ref
SrAl_2_O_4_	Eu^2+^	-	Solid-state reaction (1300 °C)	Green (λ_em_ = 515 nm(295 K) λ_em_ = 445 nm(20 K))	It was found that the luminescent center is the same, but excitation processes are different at different temperatures.	[31]
			Solid-state reaction (1250 °C)	Green (λ_em_ = 512 nm)	The position of the Eu 4f states showed the charge transfer transition.	[28]
			Combustion method (600 °C)	Green (λ_ex_ = 360 nm, λ_em_ = 513 nm)	The ratio of Eu^2+^ to Eu^3+^ is changed depending on the total concentration of Eu dopants, determining the luminescence color of the phosphors.	[32]
			Combustion method using urea at 500 °C and calcinated at 1000 °C	Green (λ_em_ = 520 nm)	The luminescence mechanism and temperature dependence of bands intensities are discussed on the crystal field theory and the vibronic approach.	[7]
	Dy^3+^	-	Combustion method (600 °C)	λ_ex_ = 356 nm, λ_em_ = 480 nm, 573 nm, 670 nm	The piezo-electricity was suggested to be responsible for producing mechanoluminescence in prepared phosphor.	[27]
	Tb^3+^		Precursor route via the thermal decomposition of tartarate compounds	λ_em_ = 542 nm	They demonstrated that the precursor method via the thermal decomposition of multimetallic tartarate compounds is a quick, simple and inexpensive way for the preparation of alkaline-earth aluminate powder.	[33]
	Eu^2+^ or Ce^3+^	-	Combustion method (600 °C)	Eu^2+^:λ_ex_ = 230, 350 nm, λ_em_ = 498 nm, Ce^3+^:λ_ex_ = 266, 331 nm, λ_em_ = 371 nm	Experimental results matched with the predictions of Dorenbos’ model.	[30]
	Eu^2+^ or Nd^3+^	-	Solid-state reaction (1000 °C)	-	The structures of the alkaline earth aluminates were systematically studied using a combination of synchrotron X-ray and neutron powder diffraction.	[13]
	Eu^2+^	Dy^3+^	Solid-state reaction (1300 °C)	Green (λ_ex_ = 365 nm, λ_em_ = 520 nm)	They observed that Dy^3+^ ion creates the highly dense trapping level by acting as the hole-trap.	[5]
			Floating zone technique	Green (λ_em_ = 520 nm)	The intensities and the persistent times of the phosphorescences are found to depend on the growth atmosphere.	[34]
			Laser-heated pedestal growth method	Green (λ_em_ = 520 nm)	It was found that multiple trapping centers are involved in the phosphorescence dynamic processes, which are responsible for the long persistence.	[35]
			Solid-state reaction (900–1350 °C)	λ_em_ = 518 nm	The depth of Dy^3+^ trap levels is in the order of BaAl_2_O_4_ host > CaAl_2_O_4_ host > SrAl_2_O_4_ host.	[36]
			Sol–gel method (900–1250 °C)	Green-blue (λ_em_ = 511 nm)	It was found that the single-phase SrAl_2_O_4_ was formed at 900 °C, which is 300 °C lower than the required temperature for the conventional solid-state reaction.	[37]
			Combustion method (600 °C)	Yellow-green (λ_em_ = 516 nm)	They proposed that phosphor samples obtain a persistent luminescence with the aid of the energy transfer at the trap level.	[38]
			Combustion method (500 °C)	λ_em_ = 528 nm	They found that the monoclinic crystal structures of both CaAl_2_O_4_ and SrAl_2_O_4_ are more appropriate in creating the traps, which is directly related to the long afterglow phenomena.	[39]
			Laser synthesis	Green (λ_em_ = 520 nm)	This laser melting method is a promising route for the synthesis of ceramic phosphors.	[40]
			Solid-state reaction (~1300 °C)	Green (λ_em_ = 520 nm)	A systematic investigation of the composition of phosphors, such as the concentrations of Eu^2+^,Dy^3+^, alkali metal, alkaline earth metal, Si ions.	[12]
			Flame spray pyrolysis technique	Green (λ_em_ = 525 nm)	The flame spray pyrolysis technique was demonstrated to manufacture the rounded and spherical particles of SrAl_2_O_4_:Eu^2+^/Dy^3+^ phosphor without any problem.	[41]
		Nd^3+^	Combustion method (550 °C)	Green-blue (λ_ex_ = 280 nm, λ_em_ = 612 nm)	Nd^3+^ trap levels can be thought of as the lanthanide element that causes long phosphorescence at room temperature.	[42]
		Na^+^	Solid-state reaction followed by ball-milling	Green (λ_em_ = 520 nm)	This report presents the factors affecting the luminescence properties of the Eu^2+^-, R^3+^-doped SrAl_2_O_4_.	[43]
		Dy^3+^ or Nd^3+^	Combustion method followed by annealing at 1150 °C	Green (Dy^3+^: λ_em_ = 515 nm, Nd^3+^: λ_em_ = 480 nm)	Eu^2+^ photoluminescence is observed to be shifted in a monoclinic/orthorhombic structure.	[44]
		Dy^3+^,Tb^3+^	Combustion method (600 °C)	Green (λ_em_ = 513 nm)	Compared with SrAl_2_O_4_:Eu^2+^,Dy^3+^ phosphor, the initial luminescence brightness improved, and the long afterglow time was prolonged in SrAl_2_O_4_:Eu^2+^, Tb^3+^ phosphor.	[45]
		La^3+^–Lu^3+^, Y^3+^; excluding Pm^3+^ and Eu^3+^	Solid-state reaction (1250–1300 °C)	Green (λ_em_ = 520 nm)	The co-doping by Dy^3+^ intensifies the luminescence by an order of magnitude, whereas the easily reducible rare earths, such as Sm^3+^ and Yb^3+^, suppressed both the afterglow and the thermoluminescence.	[46]
Sr_4_Al_14_O_25_	Eu^2+^	Dy^3+^, Er^3+^	Solid-state reaction (1300 °C)	Green-blue (λ_em_ = 481, 492 nm, and 529 nm)	Appropriate Er^3+^ doping significantly enhanced the afterglow performance of the phosphors, but excessive Er^3+^ doping caused concentration quenching.	[47]
Sr_4_Al_2_O_7_	Eu^3+^, Eu^2+^	-	Solid-state reaction (1500 °C)	Red (λ_ex_ = 450 nm, λ_em_ = 607 nm)	Sr_4_Al_2_O_7_ has higher emission intensity than Sr_3_Al_2_O_6_ due to the higher optimum doping concentration of Sr_4_Al_2_O_7_ phosphor.	[31]
	Eu^2+^	Ca^2+^	Halide-assisted solid-state reaction (1450 °C)	Red (λ_em_ = 610 nm)	Partial substitution of Sr^2+^ by Ca^2+^ in Sr_4_Al_2_O_7_:Eu phosphors is found to be an efficient way to increase the proportion of longer wavelength emission and luminescence intensity.	[48]
SrAl_12_O_19_	Eu^2+^	-	Combustion method (500 °C)	Red (λ_ex_ = 341 nm,λ_em_ = 397 nm)	Europium ions were found to be present both in divalent as well as trivalent oxidation states in the sample, and Eu^2+^ was observed as the dominant luminescent site.	[49]
SrAl_2_O_4_, Sr_4_Al_14_O_25_	Eu^2+^	Dy^3+^	Solid-state reaction followed by ball-milling	Green (SrAl_2_O_4_), Blue (Sr_4_Al_14_O_25_)	The significant loss of luminescence was observed below 2 μm average crystallite size, and performance could be partially restored by reductive annealing above 1000 °C.	[50]
Sr_3_Al_2_O_6_, SrAl_2_O_4_, Sr_4_Al_14_O_25_	Eu^2+^	-	Solid-state reaction (1350 °C)	Sr_3_Al_2_O_6_:Eu^2+^: λ_em_ = 510 nm Sr_4_Al_14_O_25_:Eu^2+^: λ_em_ = 483 nm	The influences of Al/Sr ratio, sintering temperature, the doping concentration of europium ions on structural transformation and luminescent properties of the phosphors were studied.	[51]
SrAl_2_O_4_, Sr_4_Al_4_O_10_, Sr_3_Al_2_O_6_	Ce^3+^	-	Sol–gel synthesis (700–1200 °C)	SrAl_2_O_4_:Ce: λ_ex_ = 575~700 nm Sr_3_Al_2_O_6_:Ce: λ_ex_ = 585~675 nm Sr_4_Al_4_O_10_:Ce: λ_ex_ = 615 nm	The optical reflectance spectra clearly showed the influence of the strontium aluminate matrix on the optical properties of the synthesized phosphors.	[52]
SrAl_2_O_4_, SrAl_4_O_7_, SrAl_12_O_19_, Sr_4_Al_14_O_25_	Eu^2+^	-	Solid-state reaction (SrAl_12_O_19_:1300 °C, Sr_4_Al_14_O_25_:1400 °C, SrAl_2_O_4_:1350 °C) Citric acid method (SrAl_4_O_7_: 1050 °C)	SrAl_12_O_19_: λ_em_ = 397 nm SrAl_4_O_7_: λ_em_ = 470 nm Sr_4_Al_14_O_25_: λ_em_ = 490 nm	The Eu^2+^ emission spectra in the other aluminates showed the trend that the Eu^2+^ emission shifts to longer wavelengths with an increasing Sr/Al ratio.	[53]

**Table 2:** Comparison of reported studies for the synthesis of lanthanide-doped calcium aluminate phosphors.

Host Material	Activator	Co-Activator	Synthesis Method	Color or λ	Remarks	Ref
CaAl_2_O_4_	Eu^2+^	-	Solid-state reaction (1250–1300 °C)	Blue (λ_em_ = 440 nm)	The new mechanism was proposed, which involves the excited state absorption of two 530 nm photons via deep traps followed by trapping of electrons in shallow traps.	[75]
		-	Solid-state reaction (1300 °C)	Blue (λ_em_ = 442 nm)	Good morphology and the best luminous intensity could be gained when H_3_BO_3_ mass ratio was 0.5 wt%.	[78]
	Tb^3+^		Precursor route via the thermal decomposition of tartarate compounds	λ_em_ = 542 nm	They demonstrated that the precursor method via the thermal decomposition of multimetallic tartarate compounds is a quick, simple and inexpensive way for the preparation of alkaline-earth aluminate powder.	[33]
	Pr^2+^	-	Sol–gel method	λ_em_ = 390 nm, 520 nm, 790 nm	The interlinked small granular structured particles finally formed bigger particles.	[9]
	Eu^2+^ or Nd^3+^	-	Solid-state reaction (1000 °C)	-	A systematic study of the structures of the alkaline earth aluminates using a combination of synchrotron X-ray and neutron powder diffraction.	[13]
	La^3+^ or Tb^3+^	-	Sol–gel method	Blue-green λ_em_ = 395 nm, 535 nm	Emission peak position is not altered by doping with La^3+^, Tb^3+^, but variation in the intensity is observed.	[79]
	Eu^2+^ or Ce^3+^	-	Combustion method (600 °C)	Eu^2+^:λ_ex_ = 275, 329 nm, λ_em_ = 440 nm Ce^3+^:λ_ex_ = 247, 300 nm, λ_em_ = 370 nm	Experimental results matched with the predictions of Dorenbos’ model.	[30]
	Eu^2+^	Nd^3+^	Floating zone technique	Blue (λ_em_ = 450 nm)	The intensities and the persistent times of the phosphorescences are found to depend on the growth atmosphere.	[34]
			Laser-heated pedestal growth method	Blue (λ_em_ = 445 nm)	It was found that multiple trapping centers are involved in the phosphorescence dynamic processes, which is responsible for the long persistence.	[35]
			Combustion method	Blue (λ_em_ = 440 nm)	Eu^2+^, Nd^3+^ co-doped calcium aluminate showed bright phosphorescence with a long duration.	[80]
			Solid-state reaction (1300 °C)	Blue (λ_em_ = 442 nm)	The composition of the activator Eu^2+^ and the co-activator Nd^3+^, the doping conditions with alkaline earth metals, alkali metals, and Si were optimized.	[81]
			Combustion method (550 °C)	Blue (λ_ex_ = 355 nm, λ_em_ = 492 nm)	Nd^3+^ trap levels can be thought of as the lanthanide element that causes long phosphorescence at room temperature.	[42]
		Dy^3+^	Solid-state reaction (900–1350 °C)	λ_em_ = 445 nm	The depth of Dy^3+^ trap levels is in the order of BaAl_2_O_4_ host > CaAl_2_O_4_ host > SrAl_2_O_4_ host.	[36]
			Combustion method (500 °C)	λ_em_ = 449 nm	They found that the monoclinic crystal structures of both CaAl_2_O_4_ and SrAl_2_O_4_ are more appropriate in creating the traps, which is directly related to the long afterglow phenomena.	[39]
		Na^+^	Solid-state reaction followed by ball-milling	λ_em_ = 440 nm,	This report presents the factors affecting the luminescence properties of the Eu^2+^-, R^3+^-doped SrAl_2_O_4_.	[43]
		La^3+^	Combustion method (600 °C)	blue-purple (λ_em_ = 440 nm)	They proposed that phosphor samples obtain a persistent luminescence with the aid of the energy transfer at the trap level.	[38]
		Dy^3+^, Nd^3+^, La^3+^	Solid-state reaction (1380 °C)	Blue (λ_em_ = 440 nm)	Both initial brightness and persistent afterglow time of CaAl_2_O_4_: Eu^2+^, Nd^3+^ is better than those of CaAl_2_O_4_: Eu^2+^,Dy^3+^, and CaAl_2_O_4_: Eu^2+^, La^3+^.	[82]
		La^3+^–Lu^3+^, Y^3+^; except Pm^3+^, Eu^3+^	Solid-state reaction (1250–1300 °C)	Green (λ_em_ = 440 nm)	The co-doping by Dy^3+^ intensifies the luminescence by an order of magnitude, whereas the easily reducible rare earths, such as Sm^3+^ and Yb^3+^, suppressed both the afterglow and the thermoluminescence.	[46]

**Table 3:** Comparison of reported studies for the synthesis of lanthanide-doped barium aluminate phosphors.

Host Material	Activator	Co-Activator	Synthesis Method	Color or λ	Remarks	Ref
BaAl_2_O_4_	Eu^2+^	-	Solid-state reaction (1400 °C)	λ_ex_ = 340 nm, λ_em_ = 498 nm	The Eu^3+^ reduction in BaAl_2_O_4_:Eu^2+^ prepared in the air could be explained with the charge compensation model.	[8]
	Ce^3+^	-	Solid-state reaction (900–1350 °C)	λ_ex_ = 357 nm, 335 nm λ_em_ = 450 nm, 402 nm	Site-selective thermoluminescence spectra showed that traps were close to the corresponding Ce^3+^ ion.	[88]
	Cr^3+^	-	Combustion method (500 °C)	Red (λ_em_ = 705 nm)	The site symmetry of Cr^3+^ ion in this phosphor is responsible for a distorted octahedron.	[89]
			Hydrothermal route followed by a thermal treatment	-	The dopant Cr^3+^ cations increased lattice strain and disturbed the crystallites to grow by acting as defects in the barium aluminate structure.	[90]
	Eu^2+^ or Nd^3+^	-	Solid-state reaction (1000 °C)	-	A systematic study of the structures of the alkaline earth aluminates using a combination of synchrotron X-ray and neutron powder diffraction.	[13]
	Eu^2+^ or Ce^3+^	-	Combustion method (600 °C)	Eu^2+^:λ_ex_ = 270, 328, 397 nm, λ_em_ = 485 nm, Ce^3+^:λ_ex_ = 246, 292, 308 nm, λ_em_ = 386 nm	Experimental results matched well with the predictions of Dorenbos’ model.	[30]
	Eu^2+^	Dy^3+^	Solid-state reaction (900–1350 °C)	λ_em_ = 496 nm	The depth of Dy^3+^ trap levels is in the order of BaAl_2_O_4_ host > CaAl_2_O_4_ host > SrAl_2_O_4_ host.	[36]
			Solid-state reaction (700–1500 °C)	Green-blue (λ_em_ = 500 nm)	The dopant (Eu^2+^) and co-dopant (Dy^3+^) concentrations affect the crystallinity and luminescence properties of the materials.	[87]
			Combustion method (500 °C)	λ_em_ = 450 nm	The hexagonal structure of BaAl_2_O_4_ can only produce shallow traps, resulting in a short afterglow.	[39]
			Combustion method (400–600 °C) or Solid-state reaction (1500 °C)	λ_em_ = 505 nm	They found that the method of preparation has a significant effect on the defect structure of the materials.	[87]
			Combustion synthesis method assisted by microwave irradiation	Blue-green (λ_em_ = 496 nm)	The surface of the BaAl_2_O_4_:Eu^2+^,Dy^3+^ powder samples showed lots of voids and pores.	[91]
			Solid-state reaction (1300 °C)	Green (λ_ex_ = 355 nm,λ_em_ = 499 nm)	The photoluminescence efficiency increased with increasing Eu^2+^ concentration until 3 mol% then decreased at higher concentrations due to the concentration quenching effect.	[92]
			Combustion method (500 °C)	Blue–green (λ_ex_ = 340 nm, λ_em_ = 505 nm)	The powders exhibited high initial brightness luminescence with subdued long afterglow characteristics.	[93]
		Nd^3+^	Combustion method (600 °C)	Green-blue (λ_em_ = 500 nm)	They proposed that phosphor samples obtain a lifetime of persistent luminescence with the aid of the energy transfer at the trap level.	[38]
			Combustion method (550 °C)	Blue (λ_ex_ = 355 nm, λ_em_ = 495 nm)	Nd^3+^ trap levels can be thought of as the lanthanide element that causes long phosphorescence at room temperature.	[42]
		Cr^3+^	Solid-state reaction (1300 °C)	-	Fibre shaped morphology of the grown material was formed with sharp surface morphology like single crystals.	[94]
		Dy^3+^, Nd^3+^, Gd^3+^, Sm^3+^, Ce^3+^, Er^3+^, Pr^3+^ and Tb^3+^	Combustion method (600 °C)	Blue-green (λ_em_ = 500 nm)	The highest intensity was observed from Er^3+^ co-doping, whereas the longest afterglow was observed from Nd^3+^ followed by Dy^3+^ co-doping.	[95]
